# Supplementary material for: SPATS1 (spermatogenesis-associated, serine-rich 1) is not essential for spermatogenesis and fertility in mouse
Source: PLoS One. 2021 May 4;16(5):e0251028. doi: 10.1371/journal.pone.0251028 (PMC8096103; doi:10.1371/journal.pone.0251028)
Supplement: S1 Table — (PDF) [file pone.0251028.s001.pdf]

S1 Table. Primers used in this study.

Gene	Forward primer	Reverse primer	Product size	Use
Spats1-E3	GCCAGTCCTGTTGGATGAGG	ATGGCTCTCACATGAAGGCAC	465	Standard PCR, genotyping
Lef1	AAATGGGTCCCTTTCTCCAC	TCGTCGCTGTAGGTGATGAG	108	qRT-PCR
Tcf1	CCATCCTTGATGCTGGGATC	CTTCTCTGCCTTGGGTTCTG	141	qRT-PCR
Ccnd1	CGTACCCTGACACCAATCTC	TCTTCGCACTTCTGCTCCTC	178	qRT-PCR
c-Myc	TCCTGTACCTCGTCCGATTC	GGTTTGCCTCTTCTCCACAG	195	qRT-PCR
Dvl2	CAGTGAGCTGGAGAGTACC	TGTTGAGCGTGACCGTGATG	216	qRT-PCR
Dvl1	CTATGGTACGAGTCCCTGCTC	AGCCAGTCCACCACATCC	234	qRT-PCR
Ctnnb1	GGTGTCTGCCATTGTACG	TCCTTCCTGATGGAGCAG	223	qRT-PCR
Wnt4	GAGGAGTGCCAATACCAGTTC	ATAGGCGATGTTGTCCGAGC	258	qRT-PCR
Ppp1cc	CATATCTTGAGTGGTGCTTCA	GACAGCATCATCCAACGGCT	158	qRT-PCR
Tax1bp1	AGTGTGCATTAGGAAGGTAACT	CTACGCTGAGAGGCAGTGG	176	qRT-PCR
