# Supplementary material for: SPATS1 (spermatogenesis-associated, serine-rich 1) is not essential for spermatogenesis and fertility in mouse
Source: PLoS One. 2021 May 4;16(5):e0251028. doi: 10.1371/journal.pone.0251028 (PMC8096103; doi:10.1371/journal.pone.0251028)
Supplement: S2 Table — (PDF) [file pone.0251028.s002.pdf]

S2 Table - Quantification of the different cell populations in testicular cell suspensions from WT, *Spats1*$^{+/-}$ and *Spats1*$^{-/-}$ mice, by FCM.

	**Relative percentage of testicular cell populations with different DNA content**							
	**4C population**	**Average ± SD**	**2C population**	**Average ± SD**	**1C population**	**Average ± SD**	**Apparently sub-C population***	**Average ± SD**
WT 1	6.6	6.0±0.9	12.8	9.6±3.4	46.6	42.8±3.5	34.0	41.6±7.6
WT 2	6.7		11.5		45.0		36.8	
WT 3	6.1		9.0		40.0		45.0	
WT 4	4.7		5.0		39.6		50.7	
Spats1$^{+/-}$ 1	7.5	6.3±1.3	10.7	11.2±1.8	42.1	41.0±2.5	39.7	41.5±3.7
Spats1$^{+/-}$ 2	7.3		13.3		38.8		40.6	
Spats1$^{+/-}$ 3	5.0		9.1		39.0		46.9	
Spats1$^{+/-}$ 4	5.5		11.8		44.0		38.7	
Spats1$^{-/-}$ 1	5.7	7.9±1.8	6.6	9.2±1.8	41.8	41.7±1.4	46.0	41.2±3.4
Spats1$^{-/-}$ 2	10.0		9.7		40.4		39.9	
Spats1$^{-/-}$ 3	7.5		10.5		41.1		40.9	
Spats1$^{-/-}$ 4	8.4		9.9		43.6		38.1	

*Spermatozoa are visualized as a sub-C population, as their tight chromatin packaging reduces the number of DNA sites available for fluorochrome binding [36,37].
