# Supplementary figures and images for: SPATS1 (spermatogenesis-associated, serine-rich 1) is not essential for spermatogenesis and fertility in mouse
Source: PLoS One. 2021 May 4;16(5):e0251028. doi: 10.1371/journal.pone.0251028 (PMC8096103; doi:10.1371/journal.pone.0251028)

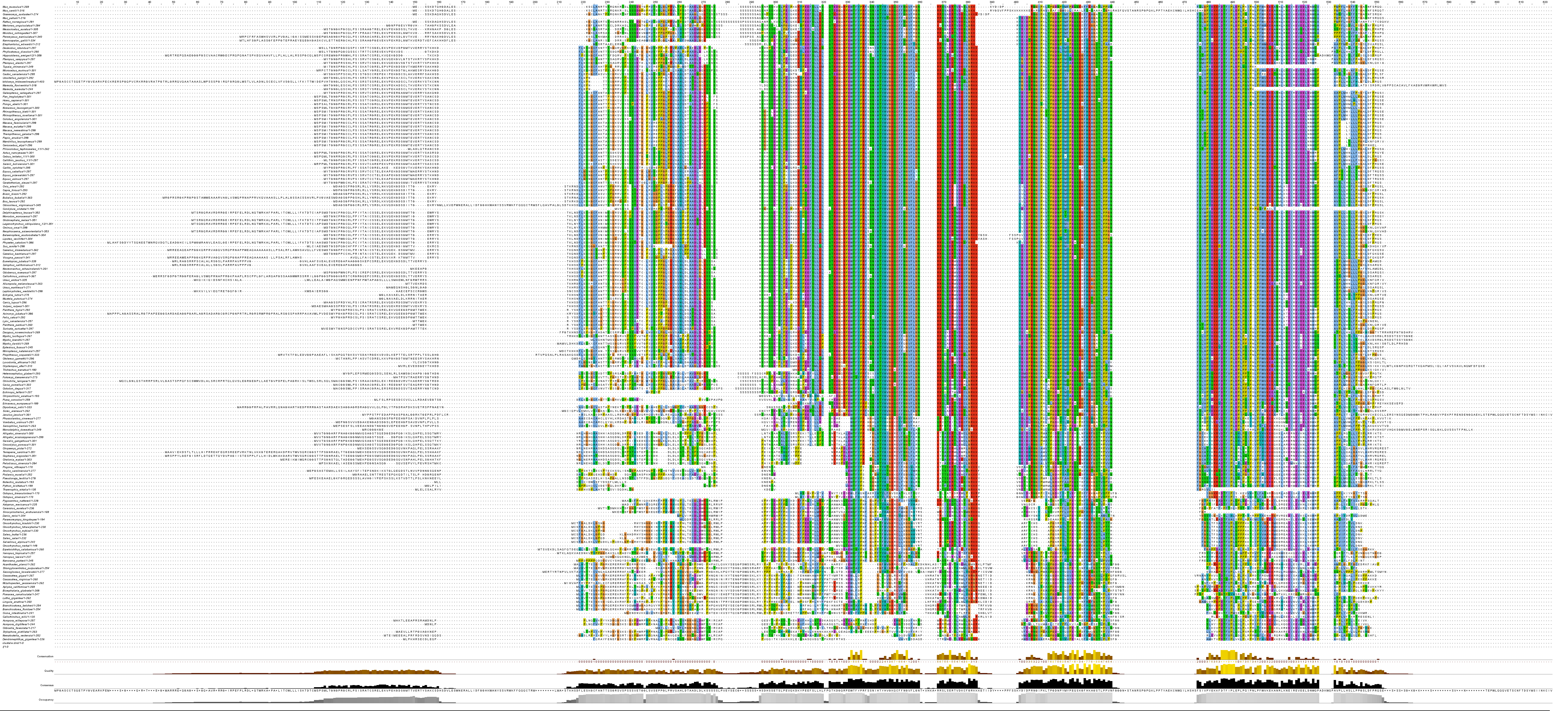

Supplement: S1 Fig — Mus musculus protein sequence was used as query. Alignment was performed with ClustalW, and visualized using Jalview 2.11.1.4 (https://mybiosoftware.com/jalview-2-6-1-multiple-alignment-editor.html). The most conserved positions along evolution (from 0 to 9) are shown below. (TIF) [file pone.0251028.s003.tif]

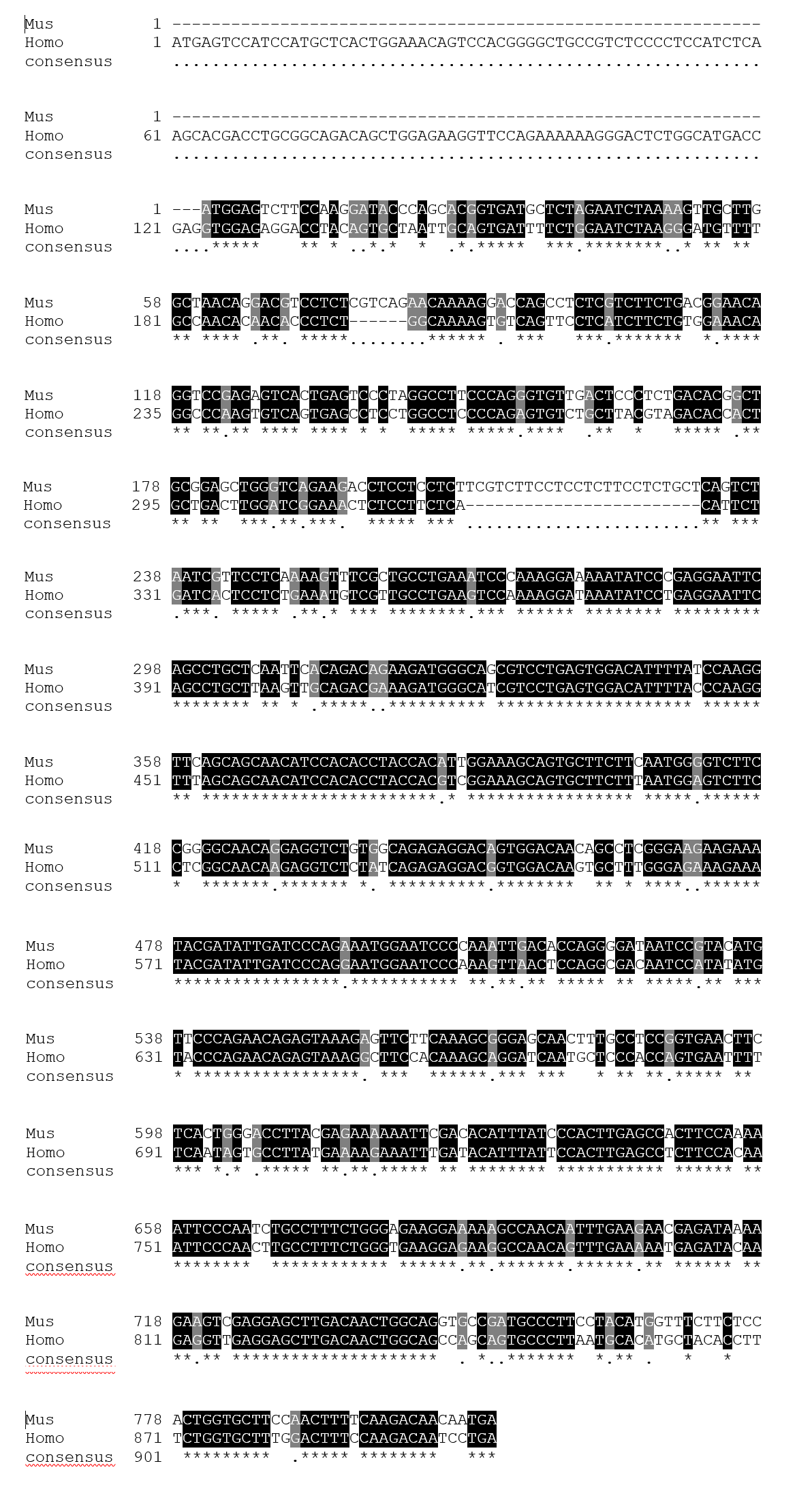

Supplement: S2 Fig — Alignment was performed with ClustalW and BoxShade. (TIF) [file pone.0251028.s004.tif]

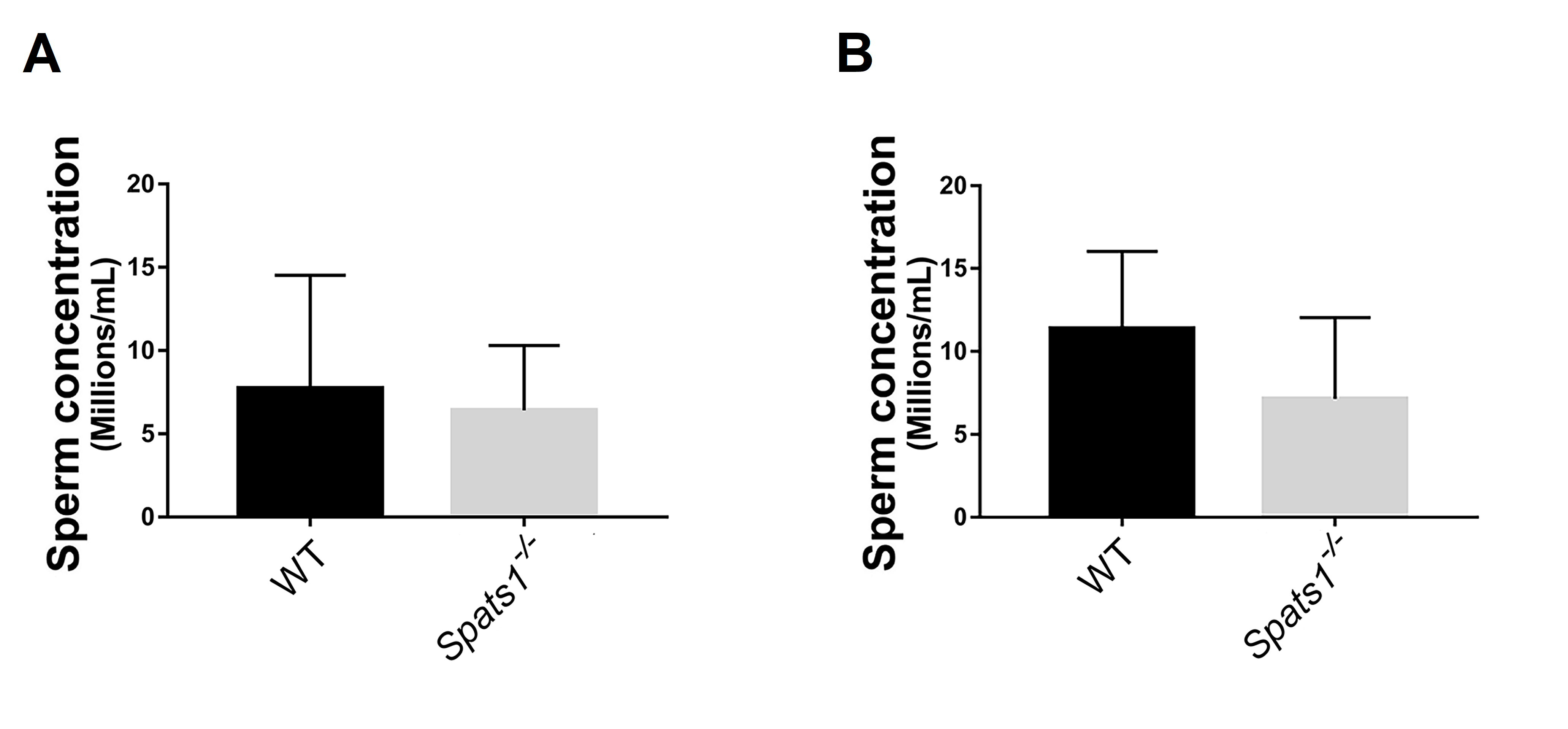

Supplement: S3 Fig — (A) 45–60 dpp animals. (B) One year old individuals. Although the count is not completely reliable in absolute numbers (as obtained through swimming out from the cauda), the data is comparable between samples. (TIF) [file pone.0251028.s005.tif]
