# Supplementary material for: SPATS1 (spermatogenesis-associated, serine-rich 1) is not essential for spermatogenesis and fertility in mouse
Source: PLoS One. 2021 May 4;16(5):e0251028. doi: 10.1371/journal.pone.0251028 (PMC8096103; doi:10.1371/journal.pone.0251028)
Supplement: S1 Raw images — (PDF) [file pone.0251028.s006.pdf]

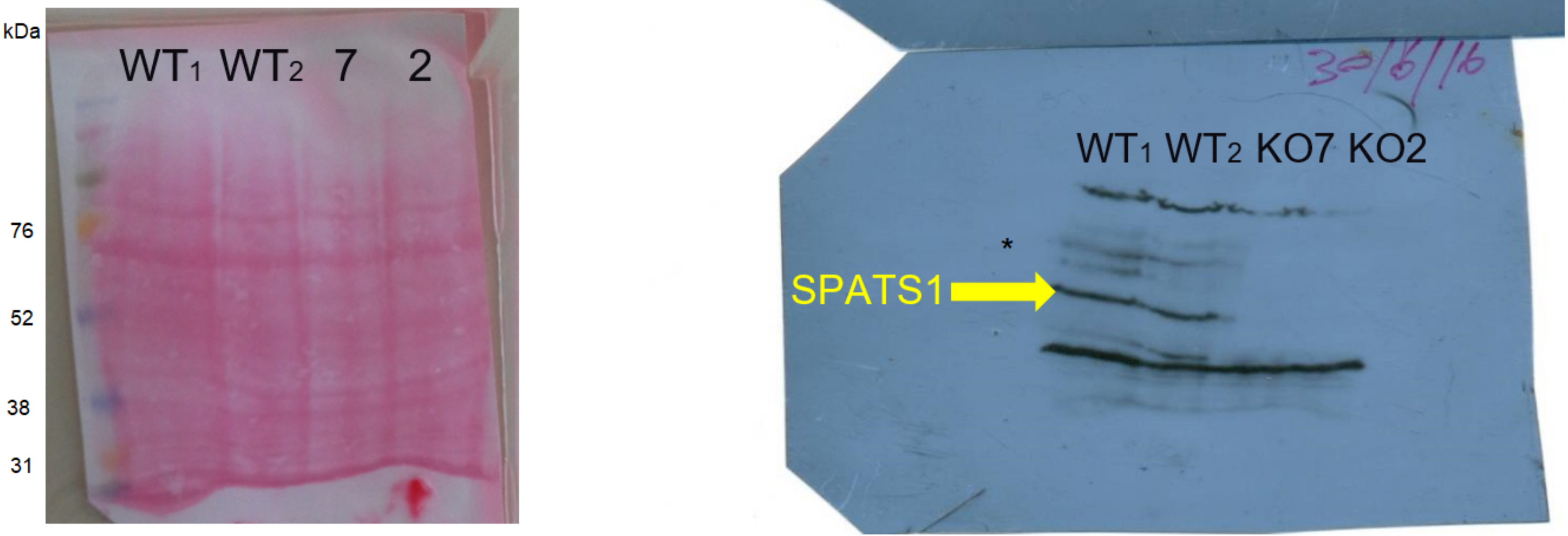

Molecular weight marker: Amersham ECL Rainbow Marker (RPN800E). Ponceau red-stained membrane (left) shows equal loading.

*Corresponds to the phosphorylated form of SPATS1.
