# Supplementary material for: SPATS1 (spermatogenesis-associated, serine-rich 1) is not essential for spermatogenesis and fertility in mouse
Source: PLoS One. 2021 May 4;16(5):e0251028. doi: 10.1371/journal.pone.0251028 (PMC8096103; doi:10.1371/journal.pone.0251028)
Supplement: Attachment — Submitted filename: RESPONSE TO REVIEWERS.docx [file pone.0251028.s007.docx]

Montevideo, April 5, 2021.-

Dear Editor,

## Thank you for considering our manuscript “SPATS1 (Spermatogenesis-associated, serine-rich 1) is not essential for spermatogenesis and fertility in mouse” ([PONE-D-21-03796] - [EMID:45ee38e7b541001a]) for publication in *PLOS ONE*. We thank all the comments and suggestions, that helped improve the ms. We are now submitting the corrected version. All the received comments from the reviewers have been considered, and we are hereby responding to all of them in a detailed fashion. Most of the suggested corrections have been included in the revised ms.

## Besides, following the journal requirements, we have provided the original uncropped and unadjusted Western blot image (S1_raw images), and amended the ethics statement to include the full name of the ethics committee that approved our specific study (now Lines 122-123 and 130-131 of the revised version with track changes).

**Editor:**

*Please include additional information on the functional role of SPATS in relation to evolutionary process. The functional relevance of the genes analysed of a particular pathway be elaborated in the discussion. Reanalyze the sperm count with tissue homogenates and using stage specific sections for histological anlyses are recommended.*

**Reply:** Thanks for the suggestions; they have been incorporated.

Specifically, as indicated, SPATS1 is evolutionarily conserved along metazoans. Now, following the recommendations from Reviewer 1, we have included the alignment of SPATS1 from mouse with that of human, and also with the predicted SPATS1 sequences from several species, from invertebrates to mammals. As stated in the Discussion, reproduction-related proteins are in general poorly conserved (most probably due to unusually fast evolution rates), and therefore this high conservation would suggest an important, conserved role for SPATS1. However, despite its high evolutionary conservation, the functional role of SPATS1 is unknown. Moreover, as shown in this ms, it is not individually essential for male fertility.

Concerning the reanalysis of sperm count, we re-analyzed the sperm count in testicular cell suspensions by flow cytometry, which represents a widely accepted means to analyze testicular cellular content, with very high quantitative analytical power and statistical weight (now S2 Table). We also replaced the histological images with matched stages as suggested. A detailed point-by-point description is in the response to reviewers below.

**Reviewer #1*:***

1) *In the abstract (i.e. last sentence) and throughout the text the authors should be careful about making parallels between Human fertility phenotypes related to SPATS1 and the mouse phenotypes described here. It is very possible – as the authors briefly note in the discussion – that one is not a good model for the other, as it is the case with many other spermatogenesis genes.*

**Reply:** Done. Thank you for the comment. We have now clarified that SPATS1 is not essential for male fertility in mouse (see lines 45 and 95 of the revised version with track changes). Besides, although previously stated, we further emphasized that the studies that had suggested a link with male testicular health were from human and bull (line 539).

*The gene is present, that seems clear. Yet, considering the scope of the study, I would recommend showing an alignment of mouse and human SPATS1 sequences (CDS and Proteins) to point to the regions that are indeed “conserved”. Please also carefully consider the next comments regarding SPATS1 evolution in the following points.*

**Reply:** Done. Following the reviewer´s suggestion, in the revised version we have included an alignment of mouse and human SPATS1 sequences, both for proteins (newly included Fig 1B) and for CDS (newly included S2 Fig), to show the extensive similarities. We have also payed careful attention to the related comments addressed in the following points (see below).

2) *Page 4 Line 78-80: What is the proposed function across evolution? Meiosis? Is it a sex-specific gene other metazoan?*

**Reply:** There is no clue about the possible function. Yes, it is a testis-specific gene in every species for which we could find expression-related information in databases. The reviewer is right that “supports the idea that this protein could have an important, conserved role in testis” can be interpreted as if an important role for this protein had been proposed. As the idea is based on the high conservation level (in this regard, it must be recalled that reproductive proteins are in generally poorly conserved, as stated in the Discussion section), we have now replaced “supports the idea” with “allows to suspect” (line 79).

3) *Page 12 Line 280-281: Clarify this sentence. What is the observation?*

**Reply:** Done. The wording of the sentence has been changed to clarify the sentence. We hope it is clearer now (now lines 286-290).

4) *The conclusion from figure 1 need to be developed to fully support the authors conclusions: the authors need to show an alignment or a few representative sequences to support the claim that it is “highly conserved” (again to distinguish between evolutionarily retained and conserved at the sequence level). Alternatively, they could use branch length on the tree to support this claim. Finally, the authors need to specify what type of tree is displayed and the scale used (substitutions per site maybe?) in Fig1.*

**Reply:** Done. Thank you for the suggestion. We have now included an alignment of about 170 species (newly added S1 Fig). This alignment includes most of the species that were used to make the tree from Fig 1A. Besides, in the legend to Fig 1A we have explained that the tree is indeed a substitutions per site tree (it is represented to scale, with branch lengths representing the number of substitutions per site; lines 303-304), and the scale is shown in the figure. Sorry for not clarifying it before.

5) *Page 13 line 299: What does differentially expressed means here? Relative to what? Please clarify.*

**Reply:** Sorry, the reviewer is right. *Spats1* is differentially expressed in the testis compared to other tissues. The sentence has been now reformulated to clarify this (now line 312).

6) *Can the author specify how many backcrosses in which strain background was made to produce the experimental set of mice?*

**Reply:** Three backcrosses to C57BL/6J were done. This information has been now included in the text (lines 151-152).

7) *Can the author specify where the antibody maps relative to the full-length protein and frame-shifted (truncated) sequence?*

**Reply:** This was a commercial antibody, and the exact amino acidic sequence was proprietary information from the company. Anyway, by asking the company we could find out that it spanned the central region of the protein (as stated in the manuscript), around amino acid 186. As the frameshift in the KO protein is at aa 90 (thus missing the central and C-t regions, which are the most highly conserved along evolution) this antibody would not recognize the mutant protein.

8) *Page 17 Line 412-415: maybe the author could show results of these qPCRs for 4C cells.*

**Reply:** Thank you for the suggestion. In fact, we are not sure whether the targets of the Wnt pathway should be mostly expressed in 4C cells. Although the highest SPATS1 expression levels were detected in spermatocytes (and even more so during the first spermatogenic wave), relatively high expression levels were also detected in other testicular cell types, including Sertoli cells, spermatogonia, and peritubular myoid cells. Furthermore, in embryonic testes SPATS1 is detected in gonocytes (at least in the rat).

The targets of the Wnt pathway have been detected in different testicular cell types as well. Most reports about testicular disruption of the Wnt pathway show overexpression of Wnt targets in Sertoli cells and spermatogonia (e.g. in mutants with altered β-catenin expression). On one hand, aberrant activation of Wnt signaling targets in testis disrupts Sertoli cells differentiation and their ability to support spermatogenesis, resulting in seminiferous tubule degeneration and infertility. However, the different reports are contradictory concerning the developmental moment in which this effect is observed, ranging from embryonic life to adulthood (e.g. Boyer et al., 2008, doi 10.1095/biolreprod.108.068627; Chang et al., 2008, doi 10.1242/dev.018572; Tanwar et al., 2010, doi 10.1095/biolreprod.109.079335). On the other hand, increasing evidence suggests that both disruption and overexpression of the Wnt pathway specifically in male germline also affects spermatogenesis. For instance, a study in which the Wnt pathway was overactivated in the germ line through constitutive β-catenin expression, showed overexpression of the downstream targets (*TCF1*, *Lef1*, *cyclin D1*) in germ cells, and particularly in spermatogonia (Kumar et al., 2016, doi 10.18632/oncotarget.13920). Again, there are inconsistencies concerning the exact timing (e.g. Chang et al., 2011, doi:10.1371/journal.pone.0028039; Kerr et al., 2014, doi: 10.1095/biolreprod.112.105809; Kumar et al., 2016, doi 10.18632/oncotarget.13920; Chassot et al., 2017, doi: 10.1016/j.ydbio.2017.04.010). In addition, discrepancies between in vitro and in vivo results are not uncommon (e.g. Xue et al 2021, doi 10.1093/molehr/gaaa085).

As a consequence, due to the intricate signaling within the seminiferous epithelium (e.g. between somatic and Sertoli cells), and to the complexity of Wnt signaling itself, in case the Wnt pathway would be affected by the absence of SPATS1, we cannot be sure if we should expect the effect particularly in spermatocytes or in other cell types as well. This is the reason why we decided to analyze it in whole testes.

This being said, we agree that it could be interesting to analyze the expression of Wnt targets in isolated cell types (i.e. 4C cell population, and maybe also 2C), to see if at least some subtle expression differences were detected in specific cell types. Unfortunately, due to Covid 19 pandemic, the transgenic facility significantly reduced its staff and workload, discontinuing most of the lines (including *Spats1* KO) and freezing embryos. We must also take into consideration that in testicular studies in which some significant alterations of the expression levels of the Wnt pathway targets were observed, these were usually acccompanied by dramatic phenotypic changes (alterations in testis development, infertility, and/or testicular cancer [e.g Kerr et al., 2014; Kumar et al., 2016; Lanza et al., 2016, doi: 10.1080/15384101.2016; Chassot et al., 2017]). Therefore, as *Spats1* KO mice do not present an overt phenotype, we think that the effort of reviving the mouse line to repeat the qRT-PCR assays in isolated cell populations is not worth it, as it is highly unlikely that even subtle changes in gene expression will be detected.

9) *Page 17 Line 419: please indicate here what is the expected level and function of c-Myc in WT testes.*

**Reply:** Done. We have now indicated it in the text of the revised version (now lines 439-440).

Briefly, the transcription factor/proto-oncogene *c-Myc* is one of the most common targets of the Wnt pathway that are activated by the β-catenin/TCF4 complex (He et al., 1998, doi 10.1126/science.281.5382.1509), together with *Lef1*, *Tcf1*, *cyclin D1*.

Particularly in WT testis, *c-Myc* has been shown to be expressed in Sertoli cells from 8-day-old rats but hardly detectable in cells from those aged 14 and 28 days, and a role in immature Sertoli cells has been suggested (Meroni et al 2019, doi: 10.3389/fendo.2019.00224). Interestingly, different reports have detected *c-Myc* overexpression in adult testes of mice and rats under abnormal conditions such as treatment with toxic substances (e.g. Wang et al., 2020, doi 10.1111/and.13841; Somade et al., 2020, doi: 10.1016/j.metop.2020.100051).

In summary, *c-Myc* expression is not expected to be observed in WT adult testis. As the study referred in the manuscript (using heterologous cell culture transfections) suggested that SPATS1 would be a TCF4 repressor, we aimed at finding out whether *c-Myc* (as well as other common targets) was upregulated in *Spats1* KO mice. Furthermore, since – as mentioned in the ms – a report suggested a possible link between *Spats1* mutation and human testicular cancer, we found it interesting to analyze if the expression levels of *c-Myc* (a proto-oncogene) were altered in the testes of *Spats1*-deficient mice.

10) *Page 21 Line 483: I would not be as categorical that SPATS1 is *not* a member of a multi-gene family. There is also SPATS2 and 2L in the mouse and human genome. Not to mention the SPATA gene family…*

**Reply:** Thank you for the comment. To the best of our knowledge, SPATS2 and SPATS2L do not have any sequence similarity to SPATS1. BLAST alignments indicate that: “No significant similarity was found”, neither in mouse nor in human, and neither at the protein nor at the nucleotide levels (SPATS2 and 2L have partial similarity between them, though). Besides, according to our analyses, they do not share any common domain or other common trait.

In the original paper where SPATS2 was described, and named p59^scr^ (Senoo et al., Biochem Biophys Res Commun 292:992, 2002; doi: 10.1006/bbrc.2002.6769), the authors state: “*The entire protein was rich in serine residues (13.6%), especially within amino acids between 373 and 411 (35.0%). Because of these characteristics, we named this novel protein p59scr (59 kDa, Serine-rich Spermatocytes and Round spermatid protein)*.” It is unfortunate that due to its overall serine content and testicular expression pattern, GenBank gave it a similar name to SPATS1, which may lead to confusion.

The ClustalW alignment for *Mus musculus* protein sequence between SPATS1 and SPATS2, below, shows the lack of similarity:

```
Spats1          ------------------------------------------------MESSKDTQHGDA
Spats2          MSRKQSQKDSSGFIFDLQSNTVLAQGGTFENMKEKINAVRAIVPNKSNNEIILVLQHFDN
                                                                 *     ** *

Spats1          LESKSCLANRTSSR------------------QNKRTSLSSSDGTGPRVTESLGLPRVLT
Spats2          CVDKTVQAFMEGSASEVLKEWIVTGKKKNKKKKSKPKPASEASGSAPDSSKSAPIQEEQP
                  .*:  *   .*                   :.* .. *.:.*:.*  ::*  : .  .

Spats1          PSDTAAELG---------------------------------------------------
Spats2          ASSEKGSINGYHVNGAINDAESVDSLSEGLETLSIDARELEDPEFAAAETLDRTGSVLEN
                .*.  ..:.

Spats1          ------------------QKTSSSSSSSSSSAQSNRSSKVSLPEIPK-------------
Spats2          GVSDFEPKSLTAHSISNVQQSRNAAKSLSRTTPGAQVSNLGMENVPLSSTNKKLGSNIEK
                                  *:: .::.* * :: . : *::.: ::*

Spats1          ---------------------------EKYPEEFSLLNSQTEDGQRPEWTFYPRFSSNIH
Spats2          SVKDLQRCTVSLARYRVVVKEEMDASIKKMKQAFAELQSCLMDREVALLAEMDKVKAEAM
                                           :*  : *: *:*   * : .  :   :..::

Spats1          TYHIG------------------KQCFFNGVFRGNRRSVAERTVDNSLGKKKYDIDPRNG
Spats2          EILLSRQKKAELLKKMTDVAVRMSEEQLVELRADIKHFVSERKYDEDLGRVARFTCDVET
                   :.                  .:  :  :  . :: *:**. *:.**:        :

Spats1          IPKLTPGDNPYMFPEQSKEFFKAGATLPPVNFSLGPYEKKFDTFIPLEPLPKIP------
Spats2          LKQSIDSFGQVSHPKNSYSTRSRCSLVAPVSLSG-PSDGSAASSSPDASVPSLPGANKRN
                : :   . .   .*::* .  .  : :.**.:*  * : .  :  *  .:*.:*

Spats1          NLPFWEKEKANN----------------------LKNEIKEVEELDN-------------
Spats2          CAPREASAAMTNSSDRPCQAHREVFPGNRRGGQGYRAQSQKTADPSNPGRHDSVGRYRNS
                  *   .   .*                       : : ::. : .*

Spats1          -WQVPMPFLHGFFSTGASNFSRQQ------------------------------------
Spats2          SWYSSGPRYQGVPPQAPGNAGERSRPYSAGTNGTGAISEPSPPKPSFKKGLPQRKPRASQ
                 *  . *  :*. . ...* ..:.

Spats1          ------
Spats2          AEAANS
```

Regarding the *Spata* (spermatogenesis-associated) family, despite that one of the synonymous names that appear in GenBank for human (but not for mouse) *SPATS1* is *SPATA 8*, *Spats1* is unrelated to the rest of *Spata* genes. Moreover, a tblastn search among all GenBank conducted with mouse SPATS1 sequence and excluding *Spats1* (in order to find out if there were possible matches with other sequences but *Spats1* homologous sequences) gives the message “Not significant similarity was found”, not producing any matches with SPATS2, the products of any *Spata* gene, nor any other predicted proteins in GenBank database.

**Reviewer #2:**

*Line 32 – SPATS1 loss of function or overexpression? – I note this is further fleshed out well below in lines 81-91*

**Reply:** Done. We have now clarified this in the text (line 33 in the revised manuscript with track changes).

*Line 37 – does ‘structure’ = histology?*

**Reply:** Thanks for the observation. It has been amended by replacing “structure” by “histology” (now line 38).

*Line 58 – peculiarities? Probably should just delete this word*

**Reply:** Done. It has been deleted.

*Line 130 – “testes were weighed individual after removal of the tunica?” The tunica does not need to be removed and probably would cause more artefacts than when testes are kept intact. Why was this approach used?*

**Reply:** The reviewer is right that removal of the tunica albuginea may cause some distortion. However, we are used to removing the tunica (which we do very cleanly), because we usually employ the tissue for preparing testicular cell suspensions. Besides, we wanted to get an idea of the weight of the testicular parenchyma.

*Line 163 – were the males also 45 days of age?*

**Reply:** Yes, they were also 45 days of age. It has been now clarified (now line 167).

*Line 170-172 – you could just state Spats1 KO or WT were mated with …
How long were they mated for?*

**Reply:** Done. The sentence was reformulated according to the reviewer´s suggestion, and the mating time (three months) was included (now lines 174-175).

*Line 187 – is this a hybrid between EM and basic light histology approaches?*

**Reply:** We usually prefer this method for sample preparation for microscopy because it involves a double fixation, first with glutaraldehyde and then with osmium tetroxide, thus enabling an excellent preservation of testicular histology. Besides, although in this case semi-thin sections and optical microscopy were used, the same samples can be eventually employed for thin sections in case transmission electron microscopy (TEM) would be required.

*Line 228 – how were sperm recovered? By cutting the cauda or by retrograde perfusion?*

**Reply:** Sperm were recovered by cutting the cauda. We have now explained it in the text (now line 234).

*Line 246 – typo: “autors”*

**Reply:** Sorry, thank you for noting it. We have corrected it (now line 252).

*Figure 3A – small suggestion – change the polka dot legend to show male first like in all the others*

**Reply:** Done, thank you for the suggestion.

*B – change the axis to g instead of gr – you could label these as Bi, Bii, Biii*

**Reply:** Done. gr has been replaced by g, and the different parts have been labeled as suggested.

*D – these are quite beautiful and especially the lower right cross-section. It is clear that spermatogenesis looks normal, however the tubules on lower left and right and not stage matched. If you could change the WT to match the KO or vice versa that would be more appropriate. It is much simpler probably to just do normal 5 um sections and PAS stain the testes however for a similar result.*

**Reply:** Done. Thanks for the suggestion. We have replaced the lower left image (WT), to match stage with the lower right (KO).

*E- I do not think it was stated how concentration was measured but depending on the method used to extract sperm (if the caudae were simply nicked) then counting the resulting sperm is not accurate. The error bars are large so it might be useful to omit this panel. Truly accurate counts can only be done with homogenisation of the epididymis or the cauda specifically, as other methods do not fully recover the sperm from the epididymides.*

**Reply:** A fixed number of nicks were performed in each cauda (it is now explained in Materials and Methods, line 234). Following the reviewer´s recommendation, in the revised version we have omitted this panel from Fig 3E, and from Fig 4C as well.

We agree that this method is not accurate as it does not fully recover the sperm. However, we have made exactly the same cuts in each cauda, and let the sperm swim out for exactly the same time lapse (15 min) in the same conditions and in exactly the same volume, and it was performed by the same operator. Therefore, we think that - although not accurate in absolute numbers - the data is comparable between samples. As it can be useful to show the absence of significant differences between WT and KO (the error bars are large because there were individual differences, but not between WT and KO), we included it now as a supplementary figure (S3 Fig). However, we can still remove it if the reviewer disagrees.

Besides, we re-analyzed the sperm count in testicular cell suspensions by flow cytometry, which, as stated above, represents a widely accepted means to analyze testicular cellular content, with very high quantitative analytical power and statistical weight (now S2 Table).

F – progressive motility looks fine, however, the authors could also show the total motility and replace the panel to the left with this

**Reply:** Done. We have replaced Fig 3E with a panel showing total motility.

*Line 355 – no significance and it looks very similar for head defects, so it is best to just state no difference*

**Reply:** Thanks for the suggestion. We have modified it according to the reviewer´s suggestion, indicating that no significant differences were found (now lines 368-370).

*Fig 4A – the legend could go underneath the columns*

**Reply:** Thank you for the suggestion. We tried placing the legend underneath the columns, but we got the impression that it did not look good, as it was too long (because it indicates a cross). Besides, the style is standardized with that in Fig 3A that also shows the results of fertility tests.

*C – comment as above*

**Reply:** Done. Following the reviewer´s recommendation, we have eliminated Fig 4C, and replaced it with a panel showing total motility.

*B – The tubules are not stage-matched and are decent but not as good quality as in the above figure.*

**Reply:** Done. Thank you for the suggestion. We have changed the images and replaced them with stage-matched tubules.

*Discussion:
The discussion covers all topics of the m/s. Line 455 – a few hints – could be rephrased*

**Reply:** Done. We have rephrased the sentence, by replacing “A few hints” with “Some data have allowed us to suspect…” (now line 477).

We hope that with these corrections and clarifications, the ms will now be suitable for publication in *PLOS ONE*. In case there would be any additional corrections or suggestions, please let us know.

Sincerely,

Adriana Geisinger

Corresponding autor
